# Amputation des quatre membres

**DOI:** 10.11604/pamj.2014.18.283.4945

**Published:** 2014-08-07

**Authors:** Maruis Kitembo Feruzi, Cédrick Sangwa Milindi, Mireille Kakinga Zabibu, Jules Panda Mulefu, Francois Tshilombo Katombe

**Affiliations:** 1 Service d'Orthopédie et de Traumatologie de l'Hôpital Général de Référence Jason Sendwe, Université de Lubumbashi, Lubumbashi, République Démocratique du Congo

**Keywords:** Amputation, membres, appareillage, réinsertion sociale, Amputation, limbs, equipment, social reintegration

## Abstract

Les auteurs présentent les cas d'amputation des quatre membres réalisée chez trois patients différents. Ce sont des amputations réalisées pour chaque patient au cours d'une seule hospitalisation et en un seul temps opératoire. Deux patients pour gangrène sèche infectée et un pour amputation traumatique des quatre membres. L'amputation d'urgence a été pratiquée en premier temps suivie de remodelage des moignons d'amputation en second temps. L’évolution de tous les patients a été bonne.

## Introduction

L´amputation consiste à enlever une extrémité du corps. Les causes des amputations les plus fréquentes sont vasculaires, traumatiques et tumorales [1]. Dans notre milieu, faute d'un appareillage adéquat, l'amputation d'un seul membre pose des sérieuses difficultés du point de vue esthétique, fonctionnel et psychologique. L'amputation des quatre membres, en absence d'un appareillage conséquent paraît une crucifixion du patient bien qu'elle vise à sauver la vie de celui-ci.

## Méthodes

Il s'agit d'une étude descriptive rétrospective menée dans le service d'orthopédie - traumatologie de l'Hôpital Général Provincial de Référence Janson Sendwe pendant une période allant de janvier 2010 à décembre 2013 soit 3 ans. Ont été inclus dans cette étude tous les patients admis dans ce service et ayant subi une amputation des quatre membres. Les renseignements cliniques, le traitement ainsi que l’évolution de ces patients ont constitués l'essentiel de nos observations. Trois patients ont été retenus pendant cette période d'enquête.

## Résultats

Nous présentons ici les aspects cliniques, thérapeutiques et évolutifs de trois patients amputés des quatre membres pendant notre période d’étude.

**Premier cas:** Il s'agit d'un patient âgé de 42 ans amené à l'hôpital en Novembre 2010 par des autorités politico-administratives. Il était sans logement et passait nuit à la belle étoile. Il avait présenté une gangrène sèche infectée des moitiés distales des deux avant-bras et des deux jambes avec une délimitation très nette entre les zones gangrénées et les tissus sains. Ces zones de délimitation étaient remplies de nombreux asticots, véritables agents de décapage des tissus mortifiés. Une amputation ouverte d'urgence de tous les quatre membres a été réalisé aux tiers proximaux des deux avant-bras et des deux jambes. Une association de l'amoxicilline et du métronidazole a été adjointe. Le remodelage des moignons a été réalisé dix jours plus tard après que l’évolution locale de la plaie soit très satisfaisante. Une chaise roulante a été remise au patient qui n'a bénéficié d'aucun appareillage.

**Deuxième cas:** Il s'agit d'un patient âgé de 24 ans amené à l'hôpital en Mai 2012, après un accident de circulation. Dans un état d’ébriété et se trouvant allongé dans une position non élucidée sur la voie ferrée, il a été écrasé par un train. Il a été apporté dans un état de choc hémorragique avec broiement des avant-bras et des deux fémurs. Les segments des membres ne tenaient que par des lambeaux cutanés. Une amputation d'urgence a été réalisée au tiers moyen de deux avant-bras et à l'union du tiers moyen et tiers inférieur des deux cuisses. Le remodelage des moignons a été réalisé après la radiographie des membres. Les suites post-opératoires étaient bonnes. Le patient a bénéficié par la suite d'une chaise roulante pour son déplacement.

**Troisième cas:** Il s'agit d'un patient âgé de 60 ans qui a consulté en septembre 2013, dans un tableau clinique semblable au premier à la seule différence qu'aux membres supérieurs la gangrène s'est limitée aux doigts et aux membres inférieurs, elle s'est limitée au tiers moyen des deux jambes. Les tissus étaient infectés. Le patient était dans un état d'obnubilation dû au choc infectieux. Il a été réanimé, transfusé et a reçu une association d'antibiotiques (céfotaxime et métronidazole). Une amputation ouverte au niveau des quatre membres a été réalisée en urgence. Aux membres supérieurs, l'amputation a intéressé chaque doigt séparément avec section osseuse réalisée à la partie distale des métatarsiens. Aux membres inférieurs, elle a intéressé les tiers proximaux des jambes. Le remodelage des moignons a été réalisé après trois semaines. Les suites post opératoires étaient simples et le patient a bénéficié d'une chaise roulante pour son déplacement.

## Discussion

Dans les pays à niveau de vie élevé, les causes les plus fréquentes des amputations des membres inférieurs sont les pathologies vasculaires suivies des traumatismes et des tumeurs [1]. Cependant, dans ces pays, la chirurgie de revascularisation a connu des progrès considérables. Il devient rare, en présence d'une artériopathie sévère, que l’éventualité d'une amputation se discute en première intention. Le vieillissement de la population (augmentation des artériopathies), ainsi que la diminution importante du nombre d'amputés pour les pathologies traumatiques et tumorales (progrès des traitements conservateurs) font que les pathologies vasculaires soient responsables de près de 80 à 85% des amputations [1, 2]. La pathologie vasculaire, diabète compris, y est prépondérante, puisqu'elle concerne plus de 80% des patients [3]. Chez nos deux patients ayant développé la gangrène sèche, faute de paraclinique pour poser le diagnostic, les hypothèses diagnostiques de la maladie de Léo Buerger et l'artériopathie chronique des membres ont été avancées. Pour les membres supérieurs, la cause traumatique est la plus fréquente [1, 2].

L'amputation transtibiale est la plus fréquente des amputations [1]. Tous nos patients ont subi une amputation ouverte en moins 24 heures. Le deuxième l'a subie immédiatement. La gangrène sèche en elle-même ne présente aucune urgence d'amputation. Une délimitation nette de la gangrène avec la zone saine doit être objectivée cliniquement pour éviter le saucissonnage du membre du patient. Cependant une surinfection change le décours habituel de la gangrène sèche.

La préparation psychologique de ces patients s'est avérée aisée et sans résistance au consentement personnel. Les patients se prêtent volontiers à se débarrasser de leurs membres dont ils ne peuvent supporter ni l'aspect ni l'odeur. Pour les deux patients qui ont été amenés dans un état de choc, ce consentement a été facilement obtenu auprès des membres de leur famille pour les mêmes raisons évoquées ci-haut. Il est parfois difficile d'obtenir ce consentement pour l'amputation d'un doigt que pour celle de tous les quatre membres. La gravité des lésions, le pronostic vital du patient ainsi que l’échec du premier traitement (souvent traditionnel dans notre milieu) concourent à obtenir dans un délai bref l'accord du patient ou de sa famille face à cette grave décision.

Quant à l'appareillage, à l'heure actuelle, il est inconcevable de décider d'amputer sans au préalable envisager et planifier l'appareillage du patient. La prothèse joue un rôle esthétique et fonctionnel. Une étroite collaboration entre le chirurgien et l'appareilleur (prothésiste) doit exister pour fournir au patient une prothèse adaptée [4–6]. Dans notre série, aucun patient n'a bénéficié des prothèses. Ils n'ont bénéficié que des chaises roulantes offertes par l'aumônerie de l’église catholique implantée au sein même de l'hôpital et qui apporte assistance aux personnes démunis. Ce manque de prothèse rend difficile la réinsertion sociale des amputés. Leur déplacement et tous les autres besoins vitaux dépendront désormais d'une tierce personne souvent un proche de la famille. Dans notre milieu, la plupart de ces amputés restent quémander dans la rue et demeurent sans abri. Cette situation contraste avec la vie de l'athlète français Philippe Croizon âgé de 45 ans qui a été le premier amputé des quatre membres à avoir traversé la Manche à la nage le 18 septembre 2010 [7]. Camilleri a souligné le fait qu'avec l'augmentation du nombre d'amputations dites « de sauvetage » la survie est parfois possible, mais l'appareillage est inenvisageable [1].

## Conclusion

L'amputation de quatre membres est une réalité dans le milieu hospitalier. Elle change le mode de vie du patient. Dans le milieu peu nanti, cette opération vise à sauver la vie du patient. La cause principale est vasculaire. La surinfection de la gangrène sèche justifie l'amputation en urgence. Elle est le résultat d'une consultation tardive et des soins non appropriés. Les amputés sont confrontés à un véritable problème d'appareillage. Les efforts doivent être fournis par les décideurs pour une bonne réinsertion sociale des amputés.
